# Worsening Thrombotic Complication of Atherosclerotic Plaques Due to Neutrophils Extracellular Traps: A Systematic Review

**DOI:** 10.3390/biomedicines11010113

**Published:** 2023-01-02

**Authors:** Francesco Nappi, Francesca Bellomo, Sanjeet Singh Avtaar Singh

**Affiliations:** 1 Department of Cardiac Surgery, Centre Cardiologique du Nord of Saint-Denis, 93200 Saint-Denis, France; 2 Department of Clinical and Experimental Medicine, University of Messina, 98122 Messina, Italy; 3 Department of Cardiothoracic Surgery, Royal Infirmary of Edinburgh, Edinburgh EH16 4SA, UK

**Keywords:** neutrophil extracellular trap, atherosclerosis, atherosclerotic plaques, thrombosis

## Abstract

Neutrophil extracellular traps (NETs) recently emerged as a newly recognized contributor to venous and arterial thrombosis. These strands of DNA, extruded by activated or dying neutrophils, decorated with various protein mediators, become solid-state reactors that can localize at the critical interface of blood with the intimal surface of diseased arteries alongside propagating and amplifying the regional injury. NETs thus furnish a previously unsuspected link between inflammation, innate immunity, thrombosis, oxidative stress, and cardiovascular diseases. In response to disease-relevant stimuli, neutrophils undergo a specialized series of reactions that culminate in NET formation. DNA derived from either nuclei or mitochondria can contribute to NET formation. The DNA liberated from neutrophils forms a reticular mesh that resembles morphologically a net, rendering the acronym NETs particularly appropriate. The DNA backbone of NETs not only presents intrinsic neutrophil proteins (e.g., MPO (myeloperoxidase) and various proteinases) but can congregate other proteins found in blood (e.g., tissue factor procoagulant). This systematic review discusses the current hypothesis of neutrophil biology, focusing on the triggers and mechanisms of NET formation. Furthermore, the contribution of NETs to atherosclerosis and thrombosis is extensively addressed. Again, the use of NET markers in clinical trials was considered. Ultimately, given the vast body of the published literature, we aim to integrate the experimental evidence with the growing body of clinical information relating to NET critically.

## 1. Introduction

Fucks et al. [1] further emphasized the role of neutrophils as the master cells of the innate immune system, anticipating one of the mechanisms of action of neutrophils which is the generation of neutrophil extracellular traps (NETs). Since the publication of this landmark study, research consideration for the neutrophil extracellular trap (NET) has steadily increased. Recently, it affirmed a preponderant presence in the panorama of cardiovascular biology, recognizing a crucial role of the NET in the pathogenetic mechanisms that support venous and arterial thrombosis [1,2,3,4,5,6]. The NET is composed of DNA strands squeezed out from activated or dying neutrophils, decorated with various protein mediators such as neutrophil elastase or azurocidine. The latter belongs to the serprocidin family and has the function of promoting the adhesion and transmigration of monocytes, as well as influencing the proinflammatory inclination that macrophages have among their functions. These inherent features of the NET offer further insight into a formerly unlooked-for connection between inflammation, innate immunity, thrombosis, and cardiovascular disease described by Lim et al. [5] Neutrophils have the ability to respond to an expanding range of stimuli by initiating a specialized series of reactions that culminate in the formation of the NET. DNA derived from nuclei or mitochondria can contribute to the generation of the NET. The fundamental step in the formation of the NET is linked to the release from the ionic bond constraints that hold DNA together with histones. In this way, the neutrophil spreads out the linear deoxyribonucleic acid (DNA) in the extracellular space thus constituting a reticular mesh that morphologically looks like a network which confers the acronym NET extremely suitable [7,8,9]. It was observed that the existence of mitochondrial oxidative stress can be considered as an important triggering factor specifically in human aging and more particularly in cardiovascular diseases. It was also suggested that a causal link exists between the mitochondrial oxidative stress of endogenous neutrophils and the subsequent NETosis during aging with a tendency towards a greater formation of atherosclerotic lesions [1,6,7,8]. 

Although we have learned that DNA once assembled to the proteins present within the neutrophil itself constitutes the backbone of the NET, however, this DNA can collect other circulating proteins in the blood. NET-associated neutrophil-specific proteins include MPO (myeloperoxidase), the serine proteinases of neutrophils with specific functions such as cathepsin G, neutrophil elastase, and proteinase and again the proinflammatory IL (interleukin)-1a molecule. Regarding the circulating proteins of non-neutrophilic derivation aggregated to the NET, the procoagulant tissue factor is of recognized importance because it plays a crucial role in thrombogenesis [1,2,3,4,5,6,7,8,9]. 

Several studies [10,11,12,13,14,15,16,17] described the presence of neutrophil-generated NETs in atherosclerotic lesions in mice and humans [17]. In atherosclerotic plaques studied in humans, neutrophils and NETs were isolated near atherosclerotic lesion segments rich in apoptotic smooth muscle and endothelial cells (SMCs). This typical localization implies that NETs contribute not only to plaque rupture but are differentially distributed depending on the classification of the plaque in its degree of complication [12]. Human coronary plaques recovered from subjects with CAD including 44 complicated plaques, characterized by intraplaque hemorrhages, erosions, and ruptures, as well as 20 intact plaques, were evaluated by immunohistochemistry. The presence of neutrophils with myeloperoxidase, neutrophil elastase and CD177 and of NETs with citrullinated histone-3 and PAD4 were closely evaluated highlighting some specific differences between the complicated lesions compared to the intact ones. Concerning the former, neutrophils and NETs were recorded in all recovered samples, without significant differences in their extension or in the presence of ruptures, erosions, and intraplate hemorrhages. In contrast, intact plaques did not reveal the presence of NETs. Furthermore, evidence of the fundamental importance for the evolution of atheromatous plaque in patients with CAD involved the adjacent perivascular tissue. In complicated plaques, it also contained a high number of neutrophils and NETs which was not detected in intact plaques [16].

A vast body of literature is now available to corroborate that the presence of NETs in human lesions supports their substantial involvement in promoting the process of atherothrombosis. It is true that while the strong experimental evidence has been well established, evidence for the causality of NETs in human disease remains elusive. Furthermore, it remains to be clarified whether their formation precedes or follows the initial rupture of the atherothrombotic plaque. Here, we present a systematic review that offers an update on this field that is experiencing rapid and extensive scientific speculation today. 

We discuss the fundamental principles of neutrophil biology, the triggers, and the mechanisms of NET formation. Furthermore, we focus our analysis on the contribution that NET provides to atherosclerosis and thrombosis. Recent evidence produced in the literature focused attention on the use of NETs’ markers in clinical trials which were considered for the completeness of the review. Our effort was aimed at providing a synthesis between the evidence suggested in the experimental literature and the results emerging from the large body of clinical information relating to NET. We believe the evidence dispensed here could supply a basis for further discernment on the knowledge of the NET and could help the physician–patient discussion of the risks, benefits, and expectations about the role of the NET.

## 2. Material and Methods

The authors rule that all supporting data are ready for use within the article and its online supplementary files. This systematic review complies with the Preferred Reporting Items for Systematic Reviews statement.

### 2.1. Data Sources and Systematic Literature Review

**Figure 1:**
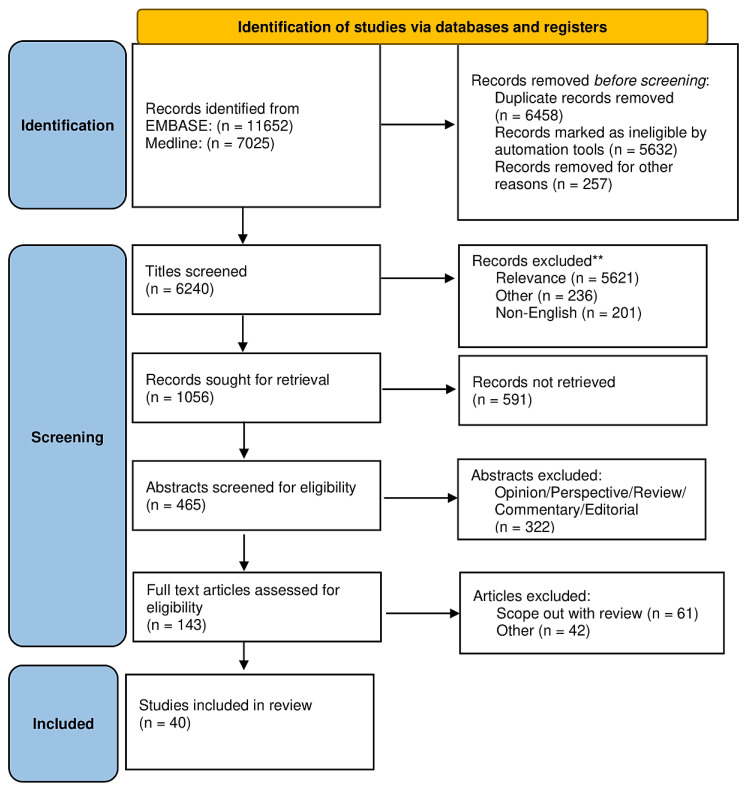
PRISMA 2021 Flow diagram for new systematic review which includes searches of database and registers only. Consider, if feasible to do so, reporting the number of records identified from each database or register searched (rather than the total number across all databases/registers). ** If automation tools were used, indicate how many records were excluded by a human and how many were excluded by automation tools. From: McKenzie et al. [18] (Supplementary Table S1).

Searches were run on 1 September 2022, to date examining Ovid’s version of MEDLINE and EMBASE in order to ensure the update of the manuscripts of interest. Inclusion criteria were English language publications, research articles, adjusted or matched observational studies or RCTs evaluating neutrophil function (15,428 to date), NETs (1628 to date), NETs’ function (746 to date), and NETosis (132 to date) and coupled with the neutrophil NET (306 to date), neutrophil NETosis (117 to date), neutrophil atherosclerosis (211 to date), neutrophil extracellular traps’ atherosclerosis (19 to date). In addition, we searched recent meta-analyses and reviews of this topic for potential additional studies. A total of 18,587 studies were retrieved, and after deduplication, 3 reviewers (S.S.A.S., F.B., and F.N.) independently screened a total of 6240 citations. All citations were reviewed by two investigators independently (S.S.A.S., F.B.), and any dissents were resolved by a third author (F.N). In case of overlapping studies, the greater series were included. A total of 465 citations were evaluated of which 40 studies met inclusion criteria and were included in the final systematic review as reported in Figure 1 including the PRISMA flow diagram. In the supplementary material, the PRISMA 2020 checklist is illustrated. The systematic review was registered in the OSF platform https://osf.io/hs2yj/, accessed on 12 December 2022.

### 2.2. Data Extraction, Quality Assessment, and Aims

Data extraction was performed independently by 2 investigators (S.S.A.S., F.B.). The following variables were included: study demographics (sample size, number of centers, publication year, study period), type of study (animal, human), aims, and major findings of the data. Only animal models, RCTs, and observational studies of high quality were included in the final analysis. The primary objective of the systematic review was, in light of recent discoveries, the evaluation of the mechanisms that determine an alteration of inflammatory responses with the release of NETs in arterial thrombosis. The secondary objective was the evaluation of the potential clinical cardiovascular implication of biomarkers of NETs and the therapeutic implication of NETs in cardiovascular conditions. The selected studies are reported in Tables 1–3.

## 3. Results

### 3.1. Recent Advances in the Biology of Neutrophils

Neutrophils are terminally differentiated cell lines with short-lived phagocyte function but with an action that is expressed in a quite unrestrained manner. To perform their function adequately, the circulating number must amount to a production of 1011 neutrophils per day in the adult human body. In medullary hematopoietic tissues, neutrophils develop and mature from progenitor cells (HSPC), and after a cascade of proliferation and differentiation phases, they are stored as a rapidly mobilizable pool. The production of neutrophils adapts to the conditions of stress when the request for their intervention supports accelerated cell production to satisfy further requests. Substantial evidence shows that hypercholesterolemia and hyperglycemia, which are considered two main actors for the stratification of cardiovascular disease risk, determine an alteration of inflammatory responses through a reprogramming of the HSPC function. The result lies in the enhancement of the subsequent myelopoiesis including the triggering of a number of granulocytes necessary to cope with the new role. Again, the proliferation towards the differentiation and functional maturation of neutrophils is evoked by the accumulation of cholesterol in the cell membrane of HSPCs. The latter mobilize even if misrepresenting their maturation towards a myeloid bias [2]. Likewise, Negardy et al. [3] reported an altered process of granulopoiesis sustained by hyperglycemia and mediated by the release of S100 calcium-binding protein A9 (S100A8/A9) from circulating neutrophils. It is important to underline that the metabolic risk factors intervene on neutrophils on two levels, both favoring an accelerated production and inducing a state of greater reactivity of the cells. Indeed, Wong et al. [4] observed that hyperglycemia is a strong conditioning factor for the production of NETs in humans and mice, through a process that requires the generation of reactive oxygen species (ROS). Therefore, the overproduction of ROS in patients with diabetes mellitus is closely related to the release of extracellular DNA from neutrophils. Similarly, the evidence reported by Tall et al. [6] suggested a substantial role of NETS in accelerating the formation of atherosclerotic lesions. This process is aided by the disgregated efflux of cholesterol in neutrophils that can also increase the activation of the inflammasome and encourage arterial infiltration of neutrophils and NETs’ release [8]. Cellular aging of HSPCs leads to somatic mutations in subjects even in the absence of overt hematological neoplasms. Substantial evidence was reported which demonstrated how HSPCs with inherent specific somatic mutations can spread out to form clones in peripheral blood according to the phenomenon known as clonal hematopoiesis. Clonal hematopoiesis primarily affects cells of the myeloid lineage, and this phenomenon is notably prone to lead to an increased risk of adverse cardiovascular outcomes [9]. 

A look at specific mutations that intervene in clonal hematopoiesis is crucial for implication in the process of the infiltration of atheromatous plaque by neutrophils. As for the genes that are most frequently mutated, they include DNA (cytosine-5)-methyltransferase 3A (DNMT3a), Ten-eleven-translocation 2 (TET2), Putative Polycomb group protein (ASXL1), and JAK2. In particular, a specific somatic signaling gain-of-function mutation in JAK2 (Janus kinase 2, V617F) activates STAT (signal transducer and activator) and deserves to be underlined. Studies on hyperlipidemia in mice by Wang et al. [10] demonstrated that *Jak2*^V617F^ in myeloid cells induces marked erythrophagocytosis associated with neutrophil infiltration. The events that emerge lead to a speeding up of the atherogenesis process associated with an increase in the characteristics of the propensity to rupture. Furthermore, two independent studies revealed that the JakV617F mutation is also coupled to spontaneous strengthening in NET release [10,11] as well as thrombus generation [11]. 

Once the acute inflammatory process has been established, the neutrophils are suddenly recalled through a well-defined recruitment cascade divided into several steps. There is no single pattern in which this process is articulated with peculiar differences that occur between tissues and with marked structural disorders that occur in the great arteries [12,13]. Several studies clarified the mechanisms that regulate the inflammatory process of atherosclerotic plaque. Drechsler et al. [14] highlighted the role of arterial neutrophil adhesion favored by platelet-producing chemokines by suggesting that this mechanism plays a substantially minor role in microcirculation. In particular, the role offered by platelet-derived Chemokine (C-C motif) ligand 5 (CCL5) and chemokine (C-X-C motif) ligand 4 (CXCL4) is crucial in activating neutrophils, especially as regards the release of the NET, thus providing a plausible explanation of the finding of the NET on the luminal side of the developing atherosclerotic lesion [15,16,17,19,20]. A relevant discovery was reported by relating the behavior of neutrophils to circadian rhythms in mice that are surprisingly interconnected. Winter et al. [21] suggested that not only an oscillation in the neutrophil count is detectable in peripheral blood during the course of the day but that the diurnal variation in the adhesion of neutrophils to the large arteries shows a 12 h phase shift compared to the same phenomenon in the microcirculation. Furthermore, recent evidence proved the existence of an intrinsic clock within neutrophils that regulates the activity of these cells including the ability to release the NET [19,22,23]. Of note, it is observed that neutrophils can invade non-inflamed tissues such as the liver and intestines through the microvasculature with a mechanism similar to that carried out by non-classical patrolling monocytes [24,25,26,27]. The point that deserves to be clarified concerns the possibility that this phenomenon also occurs in the great arteries. The anti-inflammatory offensive that can be carried out by neutrophils includes a wide range of preconstituted “weapons” which are therefore distributed in the inflammatory site. This line of attack includes the production and release of ROS as well as bioactive lipid mediators. A second offensive line is warranted by neutrophil storage of preformed granular proteins including various alarmins (e.g., cathelicidins, defensins) or serine protease and MMP-8 (matrix metalloproteinase-8). Likewise, neutrophils store high levels of MPO in the granules which can generate the avidly oxidant and chlorinating species hypochlorous acid (HOCl) that is generated in situ from sodium hypochlorite [28]. In this way, the anti-inflammatory effect is synergistically guaranteed by NADPH (nicotinamide adenine dinucleotide phosphate), associated with the surface, whose role of oxidase favors the production of superoxide anion (O2−), while myeloperoxidase, once released from the granules, induces the production of ROS with a highly oxidizing and chlorinating function such as extracellular HOCl [26,27,28]. Again, the specific function of granular proteins can be used in the inflammatory site after further degranulation and the rapid release of anti-inflammatory substances that act outside the neutrophil cells. Microbiology studies taught us a recognized and crucial antimicrobial role played by neutrophil granules. Recent findings suggested that granular proteins have an effect as important stimulant molecules on immune cells [29]. Researchers today are very actively engaged in explaining the interaction between enzymes contained in granules stored by neutrophils and NETs. In fact, when this bond is exerted, it is capable of favoring the local activity of the NETs [30,31]. The most stimulating studies are aimed at understanding how the interaction of granular proteins with the chromatin NET alters their function [2,4,32,33,34].

#### Mechanisms for the Release of NETs and Composition of NETs

The mechanism that supports the genesis of NETs is dependent on both NADPH oxidase-dependent and NADPH-independent activity. We recognize a wide range of stimuli that induce a NETosis favored by oxidase-dependent NADPH. An extrinsic stimulus involves the role of bacteria [35,36,37], as opposed to intrinsic stimuli induced by hydrogen peroxide [38,39]. Again, other stimuli that induce NADPH oxidase-dependent NETosis involve the role of concanavalin A [40] or phorbol myristate acetate [36]. Studies by Amulic et al. [40] and Hakkim et al. [41] revealed that the stimuli described above trigger the activation of downstream signaling molecules such as extracellular signal-regulated kinases (ERKs), capable of activating NADPH oxidase. It was observed that the levels of O2, CO2, bicarbonate, and pH have a modulation function on NETosis. In this regard, in the presence of phorbol myristate acetate, normoxia is not required to generate NET from a Staphylococcus aureus infection [42]. Moreover, Dölling et al. found a wide infiltration of the hypoxic heart muscle after myocardial infarction. Most of these neutrophils had viable morphology, and only a few showed signs of nuclear decondensation, a hallmark of early NET formation [43]. The stimuli work to activate NADPH oxidase which transforms molecular oxygen into superoxide. Therefore, as it was suggested, the mechanisms supporting a pharmacological inhibition of NADPH oxidase or ROS scavengers lock the genesis of the NET [41]. The superoxide is broken down into hydrogen peroxide which provides the substrate for the MPO-catalyzed production of hypochlorous acid which is a ROS with a highly oxidizing and chlorinating function. It was reported that inhibitors of the function of MPO have a substantial role in stopping the process of NETosis [44,45,46]. Two other substances of bacterial origin can induce NET formation independent of NADPH oxidase or MPO activity. These inducers are the calcium ionophore A23187 processed by Streptomyces chartreusensis and the potassium nigericin ionophore created by Streptomyces hygroscopicus [47,48].

Among the other inducers of NETosis, we recognize the nuclear peptidylarginine deiminase 4 (PAD4) which supports the conversion of positively charged arginyl residues and which are abundantly found in histones, in citrulline. The latter has the peculiar characteristic of an uncharged amino acid. The dense network of DNA and histones are tightly bound together. The intervention of PAD4 has the substantial function of breaking the ionic bonds that favor the close interconnection between the negatively charged DNA with the histones in the nucleosomes. The intervention of PAD4 leads to the release of DNA that can take place to form strands which, once extruded, generate the NETs [49,50]. Li et al. [51] suggested the key role involving PAD4 in NET formation and the killing of bacteria. The mouse animal model also revealed a strong susceptibility to bacterial infections. Evidence proven by the authors stated that NET formation depends on PAD4-mediated histone hypercitrullination. However, the role of PAD4 must be contextualized. This aspect emerges in a paper by Martinod et al. [52] who did not report increased susceptibility to bacterial infections induced by cecal ligation and puncture in PAD4-deficient mice. The formation of NETs can evade the role of PAD4 and be due to independent mechanisms which therefore do not give importance to the detection of a PAD4 deficiency as reported in a model of mice with anticitrullinated histone antibodies [53]. Although the role of PAD4 in NETosis has been downsized, however, the reduced production of NETs in PAD4-deficient mice caused by an anti-histone H3 antibody remains important evidence [54,55,56]. A landmark paper suggesting the role of circadian rhythms in the neutrophil discharge mechanism of the NET was established by Adrover et al. [57]. The authors observed that neutrophils release the NET at a certain time of day. Furthermore, they demonstrated that there is a neutrophil-intrinsic trigger, represented by the CXCR2-dependent timer that controls this rhythmicity. Together with the observed circadian oscillation of the neutrophil proteome, it was possible to demonstrate that the NET composition and therefore the functionality differ with the time of day [57]. Likewise, a marked neutrophilic degranulation activity was noted in hemodialysis patients who disclose a higher rate of mortality from bacterial infections in hemodialysis that is estimated to be 100–1000 times as compared to the healthy population. Talal et al. [58] recently described a massive neutrophil degranulation with a considerable reduction in ROS production that was noted in hemodialysis patients whose protein levels and transcriptome of neutrophils were found highly expressed. These patients experienced defective oxidative cellular signaling with a compromised function of neutrophils that exhibit a severely impaired ability to generate NETosis due to both NADPH oxidase-dependent and independent pathways, thus reflecting their loss of capacity to kill extracellular bacteria. Evidence suggested that severe and chronic impairment of NET formation led to substantial clinical vulnerability to bacteremia that most likely results from the metabolic and environmental context representative to hemodialysis patients and not by the common human genetic shortage. Importantly, aberrant gene expression and differential exocytosis of well-defined granule populations could ponder the chronic flaw in neutrophil functionality and their diminished ability to induce NETosis. These findings support the conclusion that targeting NETosis in hemodialysis patients may reduce infections, minimize their severity, and decrease the mortality rate from infections in this cohort of patients [58]. In Table 1 are reported the characteristics of more recent studies discussed.

### 3.2. The NETs Induce a Worsening of the Thrombotic Process Complicating the Atherosclerotic Plaques

**Figure 2:**
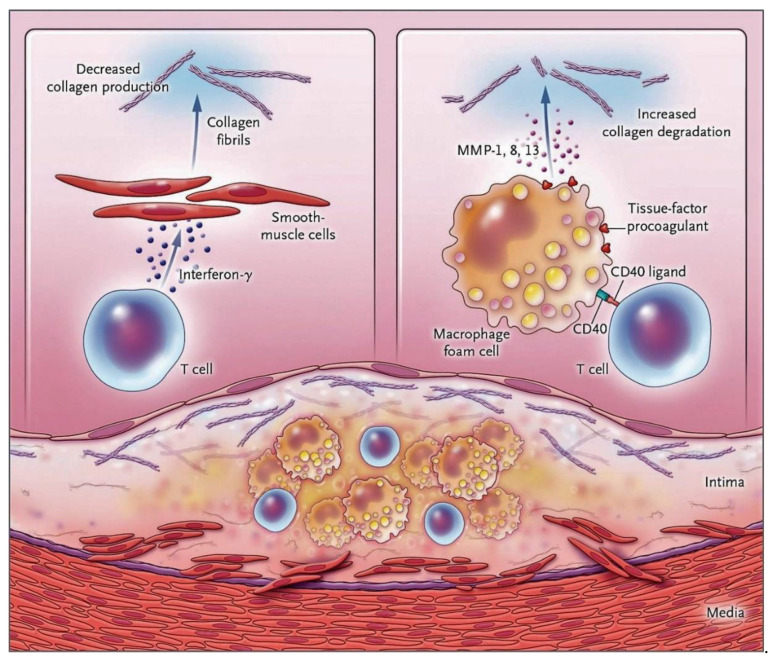
The inflammatory process intervenes in the mechanisms of rupture and thrombosis of atheromatous plaques. Mechanisms that drive plaque inflammation may accelerate and precipitate thrombotic complications of atherosclerosis by promoting symptom onset. The central lipid core of the plaque is infiltrated by foam cells of macrophages (yellow) and T cells (blue) as evidenced in the cross-section of an atheromatous plaque (bottom of figure). The cellular component of the intima and media is characterized by the presence of arterial- smooth muscle cells (red), which are producing arterial collagen, which is represented by the triple helix spiral structures. The activated T lymphocytes present in the lesion are of the type 1 helper T lymphocyte subtype with the specific function of secreting the cytokine interferon-γ, which inhibits the production of the new interstitial collagen necessary to repair and maintain the protective fibrous cap of the plaque (top left). Another T cell function leads to the activation of macrophages in the intimal lesion by expressing the CD40 ligand (CD154), which engages its cognate receptor (CD40) on the phagocyte. This inflammatory signaling results in an overproduction of interstitial collagenases consisting of matrix metalloproteinases (MMPs) that catalyze the initial rate-limiting step in collagen breakdown (top right). The ligand effect of CD40 also induces macrophages to produce more of the procoagulant than tissue factor. Thus, the inflammatory response generated in the plaque exposes the collagen in the fibrous cap to a double risk. It decreases synthesis and increases breakage, thus making the cap more susceptible to breakage. The inflammatory activation also induces an increased production of tissue factors, which trigger thrombus formation in the damaged plaque [71,72,73,74,75].

The atherosclerotic plaques are responsible for causing fatal events and are characterized by the presence of large lipid nuclei, significant infiltration of macrophages, and depletion of SMC which underlines the presence of thin fibrous caps as well as collagen exhaustion. The occurrence of fibrous cap thinning due to an imbalance between collagen formation and increased collagen degradation, caused by SMC death, can frequently be complicated by plaque rupture and the evolution to myocardial infarction with a fatal outcome [59,60,61]. In advanced lesions, an anatomopathological examination of the atheromatous plaque highlights the greater presence of infiltrates of macrophages and subgroups of T cells [62,63] compared to neutrophils which are found with a much lower frequency. However, this can be interpreted as misleading as the low counts observed at any given time may simply reflect the typical characteristic of neutrophils being present in tissue for a typically short duration. Although evidence suggests that neutrophil infiltration is poor in atheromatous injuries characterized by older lesions, clinical data support that they may play a key role in plaque complications. Although evidence suggests that neutrophil infiltration is poor in atheromas characterized by more advanced lesions, clinical data support that they may play a key role in plaque complications. Peripheral blood neutrophil counts or neutrophil/lymphocyte ratios were shown to be closely related to the risk of developing cardiovascular events and outcomes [64,65,66,67,68]. Similarly, Zhang et al. [69] demonstrated that plasma MPO levels are positively correlated with the risk of developing coronary heart disease. Plasma nucleosome levels were also observed to be associated with an increased risk of coronary stenosis, and the presence of MPO-DNA complexes supports a close link with the occurrence of major adverse cardiac events. This evidence proves that NET-derived markers can predict atherosclerotic disease charges and events [70] as reported in Figure 2.

#### Role of Nets in Plaque Destabilization: The New Challenge

An important contribution to the knowledge of the pathophysiological process that favors the progression of the atheromatous plaque and its complications was provided by the study on the infiltration of neutrophils and consequent destabilization of the atheromatous lesion. Ionita et al. [76] studied the morpho-functional alterations of the atheromatous plaque emphasizing the peculiar characteristic of the cellular infiltrate. The authors described lesions characterized by marked infiltration of neutrophils substantially present in plaques with a large lipid core. Again, they recorded an abundant concentration of macrophages and a marked modification of the vascular architecture, which was testified by a reduction in the amount of collagen fiber and SMC expression. Taken together, these alterations suggested the crucial role of neutrophils in plaque destabilization processes, with two key elements to underline, necrotic nucleus growth and thinning of the fibrous cap [76]. Given the importance of the lesions sustained by an abnormal growth of the necrotic nucleus and the thinning of the fibrous cap, Silvestre-Roig et al. [54] observed that these two anatomopathological conditions were the indication of an excessive concentration of neutrophils-triggering inflammation and in particular of the generation of NETs in promoting the destabilization of the plaque [77].

**Figure 3:**
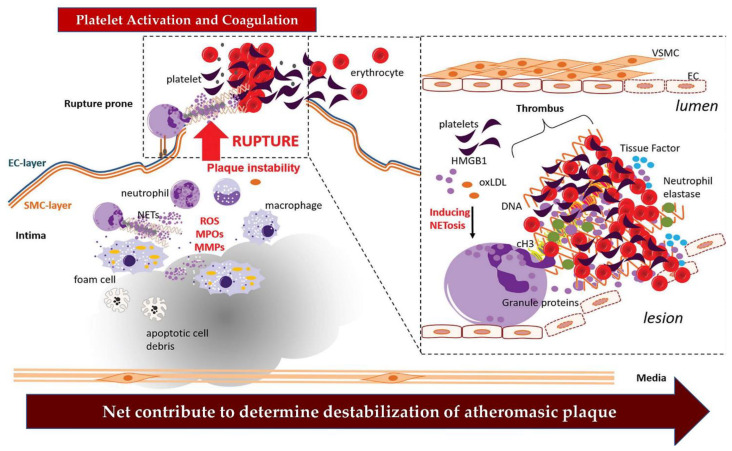
NETs’ direct arterial occlusion. During the process of atherothrombosis, neutrophil-generated NETs can intervene by supporting a series of biochemical phenomena that can ultimately trigger the activation of the coagulation cascade. Through the destabilization of the atheromatous plaque up to its rupture, NETs help to increase the stability of the thrombus. Abbreviations; EC, endothelial cell; MMPs, Matrix metalloproteinases; MPO, myeloperoxidase; ROS, reactive oxygen species; SMC, smooth muscle cell. Other abbreviations in text and tables [18,71,72,73,74,75].

Substantial evidence demonstrated a mechanical interaction between the SMCs populating the fibrous cap and neutrophils with the effect of producing their activation leading to ROS production and NET release. This sequence of events is mediated by the action of CCL7 conveyed by the SMC which supports the generation and release of NETs. Inhibition of the NET release can be achieved after treatment with a PAD inhibitor in mice with pre-existing lesions or those lacking PAD4, revealing the presence of lesions with characteristics of reduced vulnerability. The latter appears to be an expression of smaller necrotic nuclei in association with higher levels of SMC, compared with a control cohort of mice. One fact emerges with fundamental relevance in the mouse model studied and concerns the cytotoxic action exerted by NETs. This is triggered in the circuit involving the close interaction between neutrophils and NETs on the one hand and dying SMCs, dimensions of the necrotic nucleus, and structural changes typical of the thinning of the fibrous caps. Of note, the killer action of NETs on SMCs was reported in ex vivo studies which suggested that this effect was not exerted by granule-derived neutrophil proteins or the cytoplasm, but rather by a specific intervention afforded by nuclear histones. Among these, histone H4 works with well-understood cytotoxic activity, in the context of the specific action offered by histones transmitted by the NET [2,78,79,80]. Histone H4 deserves attention for its function as a strongly cationic protein, which can interact with negatively charged SMC surfaces. This bond interferes with membrane activity leading to membrane flexure and ultimately pore formation, prodromal of the lytic effect, resulting in cell death. Furthermore, the depletion of the SMC reservoir can lead to a reduction in the thickness of the fibrous cap, a phenomenon supported by marked collagen removal, which is crucial for the preservation of a suitable interstitial architecture. The demonstrated centrality of histone H4 and its involvement in the mechanisms leading to plaque rupture were suggested in further studies using a neutralizing histone H4 antibody and cyclic HIPE, exhibiting the function of histone interference peptides that are involved in targeting the N-terminus of histone H4. The two intervention strategies reported above involve the pathoanatomic characteristics of the developing atheromatous plaque and have the effect of reinforcing its stability. First, it is important to underline that in mice with advanced atherosclerosis, activation of the dsDNA sensing AIM2 inflammasome induced a marked release of the proatherogenic cytokines IL-1β and IL-18. Second, the lack or neutralization of AIM2 generated plaques that disclosed a poorer propensity to rupture; in particular, they expressed a smaller necrotic nucleus in association with a thicker fibrous cap [81,82,83] However, the exciting promises that emerged about AIM2’s role in promoting greater human plaque stability warrant further study as highlighted in Figure 3 [71,72,73,74,75].

## 4. NETs’ Actors of Arterial Thrombosis

Given the sizeable evidence recently offered, new insights into the implication of neutrophils and the role of neutrophil-derived inflammatory mediators in thrombosis have emerged [84]. The role of platelets in NET generation was discussed by Carestia et al. who highlighted the mediators, stimuli, and molecular mechanisms involved in the process of NET generation that was platelet-induced, both in human and murine models [85]. Gould et al. [86] studied the procoagulant potential of intact NETs released from activated neutrophils. The authors highlighted the relative contribution of cell-free DNA (cfDNA) and histones to thrombin generation in plasmas from patients with sepsis. A total of 1000 patients were enrolled in the DYNAMICS STUDY (DNA as a prognostic marker in ICU patients) from 2010 to 2012. The cohort included a prospectively independent population of severe sepsis patients (*n* = 400) and a broad cohort of non-septic ICU patients (*n* = 600). The first confirmation provided by the study was that NETs released by phorbyl myristate-activated neutrophils increased thrombin generation in platelet-poor plasma. This effect was closely related to DNA, after confirmation obtained from DNase treatment, and occurred through the intrinsic pathway of coagulation. In fact, in the plasma, there was a depletion of factor XII and factor XI of coagulation. In the platelet-rich plasma treated with a corn trypsin inhibitor, the addition of neutrophils activated by phorbol myristate showed an increased production of thrombin which was associated with a decreased lag time due to a mechanism dependent on toll-like receptor-2- and toll-like receptor-4-dependent. After the addition of DNase, a further increase in thrombin genesis was observed, suggesting that dismantling of the NET scaffold increases histone-mediated and platelet-dependent thrombin generation. Another key piece of evidence that emerged showed that in platelet-poor plasma samples from patients with sepsis, a positive correlation was found between endogenous cfDNA and thrombin generation, and the increase in thrombin generation was attenuated by DNAse. The results of the DYNAMICS STUDY highlighted the procoagulant activities of cfDNA and histones in the context of NETs. The study suggested the key role played by the intrinsic coagulation pathway in the pathogenesis of sepsis [86]. 

Pivotal studies by Fuchs et al. [87] and von Brul et al. [88] reported the presence of NETs associated with prothrombotic molecules such as the tissue factor, factor XII, histones H3 and H4, and fibrinogen, which promote thrombosis. Again, the NETs and the fibrin network generated by the same traps can also constitute a scaffold that entraps platelets and red blood cells. However, Noubouossie et al. [89] reported substantial evidence showing that the in vitro activation of coagulation was caused by human neutrophil DNA and histone proteins but not by neutrophil extracellular traps. Firstly, human neutrophils were incited to generate NETs in platelet-free plasma (PFP) or buffer using phorbol myristate acetate or calcium ionophore. DNA and histone proteins were also separately purified from normal human neutrophils and used to reconstitute chromatin using a salt-gradient dialysis method. Neutrophil stimulation resulted in a robust NET release. The second key finding of the analysis disclosed that in recalcified PFP, purified DNA triggered contact-dependent thrombin generation (TG) and amplified TG initiated by low concentrations of the tissue factor. Likewise, in a buffer milieu, DNA started the contact pathway and magnified thrombin-dependent factor XI activation. Recombinant human histones H3 and H4 triggered TG in recalcified human plasma in a platelet-dependent manner. Neither intact NETs, reconstituted chromatin, individual nucleosome particles, nor octameric core histones repeated any of these procoagulant effects. This study suggested that unlike DNA or individual histone proteins, human intact NETs do not directly launch or intensify coagulation in vitro. This difference is likely unfolded by the complex histone-histone and histone-DNA interactions within the nucleosome unit and higher-order supercoiled chromatin that determined the neutralization of the negative charges on polyanionic DNA leading to modification of the binding properties of individual histone proteins [89].

Accumulating evidence revealed that NETs and their components are located in human coronaries [90,91,92,93,94] as they disperse in the thrombus related to ischemic strokes [95]. This process occurs regardless of plaque type, but NETs were observed numerically to dominate more recent thrombi rather than older, more organized thrombi as demonstrated by Fucks et al. [87]. In this way, of crucial relevance are investigations of Riegger et al. [90] and de Boer et al. [92]. 

The study of Riegger et al. [90] evaluated 253 thrombus specimens from a large-scale multicenter study in patients with ST across Europe (Prevention of Late Stent Thrombosis by an Interdisciplinary Global European Effort (PRESTIGE) Investigators). Of the population studied, 79 (31.2%) patients had thrombosis with an early ST while 174 (68.8%) had a late ST. A total of 79 patients (31.2%) were treated with PCI with bare metal stenting, as opposed to 166 (65.6%) patients in whom a drug-eluting stent was used. For 8 (3.2%) PCI recipients, the thrombus examined was from a stent of an unknown type. Regarding the morphology of the thrombus samples examined, heterogeneity was observed. However, an increased abundance of platelet-rich thrombi and fibrin/fibrinogen fragments was revealed; again, 57% of the overall thrombus surface was covered with platelets. An important finding was the amount of thrombus-infiltrating leukocytes that was distinctive for both early and late ST (early: 2260 ± 1550 per mm (2) vs. late: 2485 ± 1778 per mm (2); *p* = 0.44). Of the leukocytes examined, the subset of neutrophils constituting the most important cellular component was preponderant (early: 1364 ± 923 per mm (2) vs. late: 1428 ± 1023 per mm (2); *p* = 0.81). Leukocyte counts were markedly higher than in a control group of patients with thrombus aspiration in spontaneous myocardial infarction. Extracellular neutrophil traps were found in 23% of these samples. Eosinophils were expressed as a line in all stent types, albeit the largest number was recorded in patients with late ST normalization in sirolimus and everolimus-eluting stents [90]. 

De Boer et al. [92] studied samples of different blood clots from patients who experienced acute myocardial infarction in various developmental stages. The neutrophilic cell component, NET, and immunoreactive IL-17A were preferentially localized in fresh rather than organized thrombi, thus suggesting a substantial contribution of these elements to thrombus stabilization and growth. A detailed histological analysis performed on 64 human coronary artery plaque segments included 44 complicated plaques and 20 intact plaques. In the 44 complicated plaques, where intraplaque hemorrhages, erosions, and ruptures occurred, and in the 20 intact plaques, neutrophils and NETs were most frequently observed in more recent unorganized thrombi and acute developmental intraplaque processes with ongoing hemorrhages versus older and more organized thrombi. This evidence supports the presence of NETs in distinct types of atherothrombosis, in particular, with all evidence, in fresher arterial thrombi [16,96]. The investigation of 111 different thrombi obtained from patients with acute coronary elevation syndrome reported a concentration of higher NETs, and it was correlated to a less favorable evolution of the ST elevation resolution associated with a greater extension of the infarct size. These suggest two key roles for NETs. In the former, they offer negative support to infarct lesions through the propagation of thrombosis. Instead, the second role exerted by NETs leads to the inflammation that develops distally in the infarcted myocardium and to the death of myocytes during athero-embolism. The use of DNAse, an enzyme that acts to eliminate NETs by digesting DNA strands, in regions of the infarcted myocardium, revealed a smaller extent of lesions that were related to both a reduced size of the infarction but also to a positive resolution of ST-segment elevation acute myocardial infarction (STEMI). Mangold et al. suggested that the lysis of these thrombi was accelerated by an ex vivo addition of DNAse [93]. It is important to note that observational studies performed on human samples generate intriguing hypotheses but do not allow definitive conclusions on causality to be drawn.

The role of the procoagulant tissue factor as one of the key factors for the triggering of coronary syndromes and the characteristic that they can decorate NETs was recently extensively investigated. Stakos et al. [94] studied the in vivo relevance of NETs during atherothrombosis in humans by means of selective sampling of thrombotic material. They retrieved surrounding blood from the infarct-related coronary artery (IRA) and the non- IRA during primary percutaneous revascularization in 18 patients with STEMI. Authors suggested that thrombi detached from the IRA encompassed PMNs and NETs decorated with the tissue factor (TF). The relevant findings suggested that although TF was expressed intracellularly in circulating PMNs of patients in which a STEMI occurred, however, an active TF was specifically exposed by NETs obtained from a complicated atheromatous plaque with exhibited rupture. Authors again generated NETs and subsequent TF exposure after a second necessary step consisting of the interaction between PMN and thrombin-activated platelets. The conclusions reached by Stakos et al. established that the interaction between neutrophils and platelets at the sites of plaque rupture in patients with STEMI promoted local NETs’ generation associated with the delivery of an active TF and the stimulation of prothrombotic events. The evidence highlighting this new role of NETs offers adjunctive findings to explain the mechanism by which PMNs release thrombogenic signals during atherothrombosis and may offer novel therapeutic targets [94]. 

Maugeri et al. [97] evaluated the presence of NETs in 26 retrieved thrombi from patients with acute myocardial infarction using immunohistochemistry and immunofluorescence and markers of NETs assessed in the plasma. In addition, in vitro NET generation was studied both in static and physiological flow conditions. Investigators revealed that coronary thrombi mainly consist of activated platelets, neutrophils, and NETs in close proximity to platelets. Activated platelets engaged neutrophils to NET generation. The event decreased in the presence of competitive antagonists of the high mobility group box 1 (HMGB1) protein. Hmgb1(−/−) platelets were lacking to elicit NETs, whereas the HMGB1 alone engaged neutrophils to NET generation. The integrity of the HMGB1 receptor, Receptor for Advanced Glycation End-products (RAGE), was required for NET generation, as evaluated using pharmacologic and genetic tools. Exposure to HMGB1 anticipated depletion of the mitochondrial potential that prompted autophagosome formation and extended neutrophil survival. These metabolic effects were determined by the activation of autophagy. Of note, stoppage of the autophagic flux regressed platelet HMGB1-elicited NET generation. These findings proved that activated platelets presented HMGB1 to neutrophils and engaged them in autophagy and NET generation. This chain of events may be responsible for some types of thrombo-inflammatory injuries thus designating novel tracks for molecular intervention [97]. 

We learned that optimal medical therapy for arterial thrombosis is mainly directed against platelets and coagulation factors. However, the treatment can lead to bleeding complications in an environment where the cells of the immune response and the atheromatous plaque interfere [98,99,100]. To circumvent concerns related to bleeding after treatment with antiplatelet drugs, new antithrombotic therapies, currently being studied in animals, targeted immune cells and neutrophil extracellular traps (NETs). Several studies addressed whether the immune cell composition of arterial thrombi induced in mouse models of thrombosis resembles that of human acute myocardial infarction (AMI) patients [101,102,103]. A previous report by Novotny et al. [104] studied the immune component of human thrombi with that of a mouse model. The authors evaluated 81 human arterial thrombi obtained during a percutaneous coronary procedure in patients suffering from acute myocardial infarction that were compared to arterial thrombi retrieved from mice in which experimental thrombosis was induced using ferric chloride (FeCl3) or carotid artery wire injury. The results evidenced by a detailed histological analysis demonstrated that the FeCl3-induced murine arterial thrombi and those of human coronary thrombi consisted of similar immune cells. The component of human thrombi and those withdrawn from the mouse experimental model consisted of neutrophils combined with plenty of NETs and coagulation factors. The authors found that the addition of the pharmacological administration of mice with the protein arginine deiminase (PAD)-inhibitor Cl-amidine abolished NET formation, and was related to a reduction in arterial thrombosis associated with a restriction of injury in a model of myocardial infarction. Neutrophils are a hallmark of arterial thrombi in patients suffering from acute myocardial infarction and in mouse models of arterial thrombosis. Inhibition of PAD could represent an interesting strategy for the treatment of arterial thrombosis to reduce neutrophil-associated tissue damage and improve functional outcomes. The evidence reported in Novotny’s study supports the presence of extensive neutrophil infiltration. Again, the results observed with PAD inhibition may guide the study and application of new strategies for the treatment of arterial thrombosis. The ultimate goal is aimed at obtaining a reduction in tissue damage associated with the concentration of neutrophils at the infarct site and at improving the functional outcome [104].

A convergence of findings emerges in several independent reports revealing a body of evidence supporting the substantial role that NETs play in acute ischemic stroke (AIS) and acute myocardial infarction thrombi [100]. Laridan et al. [95] investigated sixty-eight thrombi retrieved from patients who experienced an ischemic stroke and received an endovascular procedure. The analysis of thrombi was performed using immunostaining neutrophil markers such us CD66b and neutrophil elastase as well as specified NET markers such as citrullinated histone H3 (H3Cit) and extracellular DNA. For completion of the histopathology evaluation, neutrophils and NETs were quantified, and the addition of DNase 1 to the tissue plasminogen activator was used to facilitate ex vivo lysis of patient-derived thrombi. The evidence showed the marked existence of neutrophils throughout all thrombi. Again, H3Cit, a hallmark of NETs, was disclosed in nearly all thrombi. Importantly, investigators suggested that the colocalization of H3Cit with extracellular DNA released from neutrophils could be related to the peculiar occurrence of NETs in the damage site. Moreover, H3Cit was highlighted abundantly in patients with thrombi generated from a cardioembolic origin compared to other causes. Likewise, high levels of neutrophils and H3Cit were recorded in older thrombi as compared to fresh thrombi. Interestingly, using DNAse 1 added to standard t-PA, the ex vivo lysis of the patient thrombi was achieved with greater success. These findings suggest that neutrophils and NETs constitute significant components of cerebral thrombi and may offer a novel diagnostic/therapeutic pathway for targeting NETs with DNase that could potentially be used as a thrombolytic in the treatment of acute ischemic stroke [95]. 

Recently, Novotny et al. [100] performed a detailed histological analysis of arterial thrombi in which the structure of the thrombus but especially the abundance of leukocyte subsets differed between patients with acute ischemic stroke (AIS) and acute myocardial infarction (AMI). Investigators revealed that although amounts of leukocytes (*p* = 0.133) and neutrophils (*p* = 0.56) were alike between AIS and AMI thrombi, however, monocytes (*p* = 0.0052), eosinophils (*p* < 0.0001), B cells (*p* < 0.0001), and T cells (*p* < 0.0001) were found to be more abundant in patients with a stroke as to patients with AMI thrombi. In addition, the quantity and appearance of NETs revealed an inhomogeneous pattern of NETs that were experienced in 100% of patients with AIS as compared to only 20.8% of patients with AMI. The wealth of NETs in thrombi was associated with poor outcome scores in patients with AIS while patients with AMI recorded a decreased ejection fraction. This difference outlined in patients’ outcomes after AIS and AMI supports a critical impact of NETs on thrombus stability in both conditions [100]. Ducrox et al. [105] reported histological evidence in a randomized controlled trial enrolling 108 acute ischemic stroke patients from whom thrombi were recovered. The objective of the RCT was to assess the presence of NETs in thrombi retrieved during endovascular therapy in patients with AIS and their impact on tPA-induced thrombolysis. The authors demonstrated the presence of conglomerates of NETs in all thrombi with a higher concentration of networks in the outer layers of the thrombus. They concluded that the thrombus NET content was responsible for reperfusion resistance, and included both mechanistic and pharmacological approaches with intravenous tPA, regardless of their etiology. Thus, the efficacy of a strategy involving the administration of DNAse 1 in addition to tPA could be considered a new way to be explored in the context of AIS. Studies by Novotny et al. and Ducrox et al. offered an explanation of the importance of NETs in thrombosis and the clinical potential they represent. Above all, the substantial result is represented by the fact that ex vivo, recombinant DNAse 1 accelerated thrombolysis induced by the tissue plasminogen activator. However, DNAse 1 alone did not have the same effect [105].

These findings should be correlated with those suggested by Farkas et al. [91] which highlighted a difference between the content of NETs, the relative concentration of platelets, and the fibrin structure of thrombi as well as their distribution in thrombi recovered from patients with acute ischemic stroke, myocardial infarction, or peripheral artery disease. The authors reported the presence of DNA in the thrombi of acute ischemic stroke patients during the comparison, perhaps explaining why DNase 1 alone did not effectively destroy the thrombi. Furthermore, the DNA/fibrin ratio was significantly lower in thrombi recovered from patients who had experienced an acute ischemic stroke than in patients in whom thrombi were recovered from peripheral arteries. Again, the thrombi from peripheral artery disease contained fewer platelets. Bucking, thrombi from the three sites studied expressed comparable histone citrullinated levels-3 [91]. 

From these data, it emerges that the levels of DNA substantially condition the effect of the therapy. Thus, the combination of the tissue plasminogen activator and DNAse I demonstrated greater efficacy in thrombus lysis, just as it was suggested that the addition of DNAse I to DNA-poor thrombi, such as those obtained from patients with ischemic stroke, is not sufficient to lyse the thrombus. Furthermore, the presence and involvement of NETs in thrombosis may vary according to the type of artery involved. This aspect is crucial because it can direct both the type of intervention to be performed and the time necessary to obtain a benefit from the treatment. Therefore, potential therapeutic targeting should take due consideration of the age of the thrombus and the aspiration procedure which play a key role in the success of the procedure. The latter will require adaptation depending on the site and the timing of the treatment.

Studies performed in mice revealed that neutrophil-derived externalized nucleosomes associated with NETs are implicated in arterial thrombosis induced by FeCl3 application. Massemberg et al. [106] in a wild-type FeCl3-mouse model disclosed that administration of a histone-neutralizing antibody resulted in a prolonged time to occlusion associated with decreased thrombus stability in the carotid arteries. This result was not obtained after infusion of antibodies in mice with selective neutrophil elastase/cathepsin G deficiency in which vascular damage was induced. This evidence supports the substantial role played by externalized nucleosomes which allow the assembly of the complex constituted by neutrophil elastase and its substrate-tissue factor pathway inhibitor located on the surface of activated neutrophils. This mechanism may sustain the series of events leading to thrombosis. The evidence emerging in the study by Massemberg et al. supports substantial data that, in sterile inflammation that develops in large vessels, neutrophil-derived serine proteases and nucleosomes may favor the initiation of a thrombotic process responsible for myocardial infarction and heart attack stroke [106]. However, it is important to underline the independent role of neutrophils with respect to NETs which, through the release of single components, can exert stronger procoagulant effects than those induced by NETs by completing the action of the latter. This aspect emerges during in vitro activation of coagulation by human neutrophil DNA and histone proteins but not by neutrophil extracellular traps [89]. Knight et al. [102] studying an in vivo model of New Zealand mixed mice, which have the characteristic of developing a lupus-like systemic pathological condition driven by IFN type I, reported increased NETs’ formation with manifested accelerated vascular dysfunction and increased prothrombotic risk. The authors administered chloramidine to New Zealand mixed mice and recorded a substantial reduction in the generation of NETs associated with both an improvement in endothelium-dependent vasodilation and a marked reduction in the time to arterial thrombosis [102]. In a more recent study, Sorvillo et al. [107] supported the role of PAD4 in accelerating thrombus formation and stability. This process was mediated by a reduction in the clearance of von Willebrand factor (vWf) -platelet thread structures in the circulation. The proposed role of PAD4 lies in the inhibition of the vWf degrading proteinase ADAMTS-13 which acts as a disintegrin and metalloprotease with a thrombospondin-1-like domain 13 [107].

NETs are not always generated. Cherpokova et al. [108] evaluated neutrophil function in a mouse model using the specialized resolution mediator resolvin D4. The authors recorded a reduced susceptibility to NETosis induced by ionomycin administration, explaining the role of modulators exerted by these resolution mediators after administration. Their modulatory action in directing the severity of thrombo-inflammatory diseases in vivo is exerted through interference with the generation of NETs [108]. Franck et al. [109] identified a crucial role played by NETs in exacerbating the aspects of thrombosis supported by superficial erosion. Evaluations were performed on mice in which an arterial lesion was previously induced followed by a periarterial cuff-induced flow disturbance. A model thus generated allowed reproduction of the set of characteristics of human lesions complicated by superficial erosion, therefore to study the plaque complication that can previously be associated with NETs. The number of NETs generated and the initiation of thrombosis were strictly dependent on a PAD4 deficiency in myeloid cells or DNAse treatment. Either a PAD4 deficiency in bone-marrow-derived cells or administration of DNAse1 to disrupt NETs reduced the extent of arterial intimal injury in mice with arterial lesions tailored to summarize environments reproduced in human atheroma complicated by erosion. Results derived from Franck et al. suggested that PAD4 from bone-marrow-derived cells and NETs did not influence the chronic processes on the basis of induced experimental atherogenesis. In contrast, they fortuitously shared in the acute thrombotic complications of tunica intima lesions that restate features of superficial erosion [109]. The main studies identified in the systematic review are reported in Table 2.

## 5. The Cardiovascular Clinical Potential Derived from Biomarker Applications of NETs

The literature has a large number of studies in which a relationship between potential biomarkers of NETs and clinical variables emerged, including some that are discussed in the text in Table 3 [93,94,97,104,105,111]. To date, there are no solid data to support a definitive validation of these biomarkers in clinical practice, whose full consideration, therefore, remains incomplete and still open to discussion. However, the biomarkers, which several investigations adopted as reliable parameters to establish the effective involvement of NETs in the destabilization process of atheromatous plaque, are double-stranded DNA, MPO-linked DNA, citrullinated histones, neutrophil elastase, among other NET constituents in the blood or aspirated thrombi. In clinical practice, the challenge that will allow to develop and validate more specific biomarkers of NETs becomes of utmost importance. Such studies can be directed to explore different hypotheses relating to NETs’ involvement in the progression of plaque which explains this phenomenon in purely physical or deterministic terms. Furthermore, these studies can contribute to providing useful information for prognosis, and possibly could be used to designate specific therapies based on the degree of NET involvement, in the utility of personalized or precision medicine [121].

One study correlated NETs’ markers with the manifestation of more severe degrees of coronary atherosclerosis in CAD associated with stable angina as assessed by various imaging modalities [69]. Several studies suggest the presence of elevated markers of NETs in patients exhibiting acute coronary syndromes. Patients who develop CAD with ST-elevation in the course of IMA reveal an increased level of cell-free DNA and other NET constituents, compared with the control group [15,110,111,112,113,122,123]. Likewise, patients with an acute ischemic stroke disclose increased blood NET markers [114,115,124]. NET markers were observed in aspirated thrombi from patients with acute coronary syndromes, stent thrombosis, ischemic stroke, and acute peripheral arterial disease [90,91,92,93,94,95,96,97,105]. In patients with documented atherosclerosis, several clinical conditions were highlighted which may be associated with the different concentrations of NET markers. For example, NET load is closely related to infarct size as observed with magnetic resonance imaging [93,116]; it was also associated with worse outcomes at a 2-year follow-up in patients with stable coronary artery disease [117,125]. We have a growing experimental literature, which agrees with the data above, reporting that NET-derived products can be detected in systemic chronic diseases complicated by cardiovascular disease. This condition is typical of patients who associate atherosclerosis with rheumatoid arthritis [118,126]. A total of 60 maintenance hemodialysis patients were studied in a cross-sectional analysis. Of these, 30 matched for age and sex were healthy individuals and represented the negative control while the other 30 patients manifested an acute infection and represented the positive control. In the latter, a correlation was reported between the increased production of NETs associated with uremia and with a worsening of atherosclerosis disease [119]. Of no less importance is the evidence linking the formation of NETs to the vexing clinical problem of thrombotic complications associated with cancer [127]. Another example of an increase in the generation of NETs was reported in a study that enrolled 138 patients with stroke-related cancer [120]. The main studies identified in the systematic review are reported in Table 3.

## 6. Therapeutic Implications of NETs in Cardiovascular Conditions

The afforded scientific literature states that NETs contribute to thrombosis and are considered effectors in the process of the amplification of inflammation. The development of vascular damage offers additional potential for the generation of the NET and NETs as therapeutic objectives. In the course of this reported analysis, we cited numerous experimental studies that used chloramidine as an inhibitor of PAD4. While on the one hand, the use of chloramidine can be useful to demonstrate the mechanisms of interference for the formation of NET, chloramidine works to inhibit different isoforms of PAD, and for this reason, it is characterized by a lack of specificity. This constitutes an important aspect of the clinical application limiting its use. Increasing research aimed at studying other PAD4 inhibitors could provide new avenues toward a direct approach in which the goal is to limit the formation of NETs. If, on the one hand, limiting the generation of NETs can be considered a valid approach, also the acceleration of the disintegration of these reticular structures can guarantee an effective result considering another therapeutic route. It was described how DNAse-1 can dissolve NETs by breaking their DNA strands. The use of DNAse was successful when it was applied to counteract the inflammatory response of the bronchial mucosa in patients with cystic fibrosis resulting in neutrophil-rich bronchial mucosa and NET generation. Another open front for the application of DNAse in clinical therapy, supported by clinical and experimental studies, is directed towards the cure of myocardial infarction. Improvements in this area were reported in IMA injuries due to ischemia-reperfusion after DNase infusion [128,129,130,131]. As far as myeloperoxidase inhibitors are concerned, they could be effective in reducing the production of ROS associated with the generation of NETs. Furthermore, the formation of NETs can be reduced, thus limiting the harmful consequences, with the administration of drugs for preventive purposes which include colchicine, inhibitors of complement, or phosphodiesterase 4 [132,133,134].

Two randomized trials were designed for the administration of colchicine in patients with recent myocardial infarction. In the CALCOT (Colchicine Cardiovascular Outcomes Trial) study, [135] 2366 patients treated with low-dose colchicine versus 2379 placebo patients revealed a significantly lower risk of myocardial infarction [0.91 (95% CI, 0.68 to 1.21)]. Again, the hazard ratios were 0.84 (95% CI, 0.46 to 1.52) for death from cardiovascular causes, and 0.83 (95% CI, 0.25 to 2.73) for resuscitated cardiac arrest in the cohort colchicine vs. placebo. The administration of colchicine at a dose of 0.5 mg daily was associated with a significantly lower risk of ischemic cardiovascular events than the placebo in patients with recent myocardial infarction. 

Likewise in the other CONVINCE RCT (Colchicine for the prevention of vascular inflammation in non-cardioembolic patients Stroke) currently underway [136], 154 patients were enrolled from 17 countries in a multicenter international Prospective, Randomized Open-label, Blinded-Endpoint assessment (PROBE) controlled Phase 3 clinical trial. The study was designed to establish the safety and efficacy of low-dose colchicine anti-inflammatory therapy plus usual care with the goal of reducing recurrent vascular events in patients with non-severe, non-cardioembolic stroke and TIA compared with care alone. Colchicine proved safety and effectiveness as a novel therapeutic agent to induce inhibition of the inflammation cascade in patients with extra- or intracranial atherosclerosis or arteriolosclerosis, resulting in decreased vascular events [137,138]. The action exerted by colchicine could be directed towards the inhibition of inflammasome activity, [139,140,141] with the associated effect of reducing the burden of NETs generated in recent myocardial infarction or stroke tissue and could contribute strongly to support the outcome reported in the COLCOT study. Instead, the supposed action of reducing the accumulation of NETs in acute coronary syndrome suggested by the administration of anticoagulants and antiplatelet therapies provides sufficient evidence to demonstrate efficacy [142,143,144]. It is important to underline the contradiction that emerged from experimental evidence which proved how NETs can induce the polarization of macrophages in the infarct zones, promoting the healing of the affected myocardium through a push towards connectivity [145,146]. Therefore, caution in the use of NET inhibitors is necessary because their inhibition could be a sword of Damocles [144,146,147], in circumstances in which the inflammatory response can produce beneficial effects [145]. It is appropriate to affirm how the need to promote other clinical and experimental studies on the role of NETs can feed our knowledge and lead to an adequate use of drugs that interfere with the generation of NETs. 

## 7. Limitations

The initial description of NETs is relatively recent and dates back to 2004, albeit it sprouted quickly. For this reason, the current review is limited to the relative novelty of NETs even if links between data emerging in the experimental literature and those present in the clinic focus on the role of NETs in numerous cardiovascular conditions. This evidence opens new windows for the mechanistic exploration of pathophysiology. The current availability of data is also limited regarding the use of NET biomarkers that can provide a step towards personalized precision medicine capable of identifying groups of subjects particularly susceptible to certain therapies. Importantly, the limitation of the data presented could be related to the lack of manuscripts in languages other than English that were not considered for this review, which could have reduced the number of available studies. While data are abundant, exemplified by the number of hits on primary search criteria, a large proportion of these came from secondary sources (reviews and editorials) that were not included as part of the systematic review process but discussed later. Since the recognition of the role of NETs in cardiovascular diseases is of fundamental importance, even more because it identifies a new series of therapeutic targets currently under intense consideration, the analysis of data coming from randomized clinical trials registry studies including a large number of patients becomes crucial. The provenance and certainty of the data are of substantial importance to the role played by the NET in cardiovascular disease which was the subject of some criticism, reinforcing the need for rigor in basic and clinical research in this field [53].

## 8. Conclusions

Our current knowledge of NETs’ evolution and its influence on the propagation of cardiovascular insults such as acute ischemic stroke, peripheral artery disease or acute myocardial infarction, and subsequent development of heart failure are still in their infancy. However, therapeutic options look to be on the horizon. The immediate therapeutic options have the caveat of negating the beneficial effects of inflammation which play an important role in homeostasis. This should be the next avenue to the cardiovascular disease spectrum and its potential sequelae. 

## Figures and Tables

**Table 1:** Characteristics of the included studies of biology of neutrophils, NET composition, and release.

First Author/Year Ref	Type of Study	Cohort	Aims	Finding
Gu et al. (2019) Science [2]	Multicenter Center (USA/China)	Animal Model Zebrafish lines and Ldlr−/− (B6) mice	Whether AIBP orchestrates HSPC emergence from the hemogenic endothelium	AIBP-regulated Srebp2-dependent paradigm for HPSC expansion in atherosclerotic cardiovascular disease.
Lim et al. (2018) Front Immunol [5]	Prospective Multicenter Center (China/Sweden)	Blood human donors	To evaluate effects of human protease thrombin and plasmin during injury and wounding on NETome	Exogenous proteases present during wounding and inflammation influence the NETome.
Keitelman (2022) Front Immunol [7]	Prospective Multicenter Center (Argentina)	Blood human donors. Healthy vs. GOF NLRP3-mutation	To study the role of caspase-1 upon inflammasome activation to release Interleukin-1 beta (IL-1β)	Caspase-1 regulates human neutrophil IL-1β secretion.
Jaiswal et al. (2017) NEJM [9]	Prospective Multicenter Center (USA/UK/Spain)	Blood human donors/mice model Prospective CAD (4726 pt.) Retrospective Control (3529 pt.). Hypercholesterolemia-prone mice BM homozygous or heterozygous Tet2 knockout mice BM control	Association between CHIP and atherosclerotic cardiovascular disease using whole-exome sequencing	Double risk of CAD in humans with CHIP. Higher risk of atherosclerosis in mice. With higher macrophages infiltrate in Tet2 knockout mice. Higher expression of chemokine and cytokine genes.
Wang et al. (2018) Circ Res. [10]	Multicenter Center (USA/China)	Animal Model Ldlr−/− vs. BM wild-type or Mice Jak2 VF	To evaluate atherosclerosis and mechanisms in hypercholesterolemic mice with hematopoietic Jak2 VF expression	Lesion formation with increased complexity in advanced atherosclerosis hematopoietic Jak2 VF expression led. In early lesion neutrophil and macrophages infiltration.
Wolach et al. (2018) Sci Transl Med [11]	Multicenter Center (USA/Israel/UK/ Netherlands)	Human/Animal Model Human 10,000 without MPNs Mice knock-in of *Jak2*^V617F^	Whether neutrophils from patients with MPNs are triggered for NET formation	*JAK2*^V617F^ expression is linked to NET formation and thrombosis. JAK2 inhibition may reduce thrombosis in MPNs through cell-intrinsic effects on neutrophil function. PAD4 is required for NET formation increasing in *Jak2*^V617^.
Li et al. (2022) Int J Biochem Cell Biol [13]	Single Center (China)	Animal model BAP31 knockdown mice	Whether BAP31 regulates CD11b/CD18 neutrophils	BAP31 depletion exerted a protective effect on ALI. Decreased neutrophil adhesion and infiltration by blocking the expression of adhesion molecules CD11b/CD18 and PSGL-1.
Winter (2018) Cell Metab [21]	Multicenter Center (Germany/Netherlands/Spain/Sweden)	Animal model Mice Cx3cr1GFP/WTApoe−/− monocyte BM control	To evaluate that diurnal invasion of the arterial wall could sustain atherogenic growth by CCL2 and CCR2	In activity phase, chronic inflammation of large vessels nourishes on cadenced myeloid cell recruitment. Inhibition of atherosclerosis by means of pharmacological CCR2 neutralization.
Adrover (2019) Immunity [22]	Multicenter Center (Spain/USA/Germany/ France/Singapore)	Animal model. Mice engineered for constitutive neutrophil aging	To identify a neutrophil-intrinsic program that works as efficient anti-microbial defense while preserving vascular health	In mice, engineered diurnal compartmentalization of neutrophils coordinates immune defense and vascular protection becoming resistant to infection. Nevertheless, higher evidence of thrombo-inflammation and death
Yan (2021) Front Immunol [23]	Single center (USA)	Animal model C57Bl/6 mouse neutrophils containing a genomic knock-in BM control	Mutation disabling RGS protein-Gαi2 interactions (G184S) lead to worse chemoattractant receptor signaling; compromised response to inflammatory insults	Neutrophil Gαi2/RGS protein interactions limit and facilitate Gαi2 signaling. Promoting normal neutrophil trafficking, aging, and clearance.
Casanova-Acebes (2018) J Exp Med [24]	Multicenter (Spain/Singapore/Canada/Germany/ Netherlands/Japan)	Animal model	To investigate neutrophils’ capacity to infiltrate multiple tissues in the steady-state leading to process that follows tissue-specific dynamics	Homeostatic infiltration of tissues unveils a facet of neutrophil biology that sustains organ function inducing pathological states.
Zheng (2022) Proc Natl Acad Sci U S A [25]	Multicenter Center (USA)	Animal model * MISTRGGR mice	To evaluate the role of humanized mouse model MISTRGGR The mouse G-CSF was replaced with human G-CSF, and the mouse G-CSF receptor gene was deleted in existing MISTRG mice	MISTRGGR mice represent a unique mouse model that permits the study of human neutrophils in health and disease.
Amulic (2017) Dev Cell. [40]	Multicenter Center (Germany, USA)	Animal model	To investigate NETs’ formation induced by mitogens; role of phosphorylation	In neutrophils, CDK6 is required for clearance of the fungal pathogen Candida albicans. CDK4/6 is implicated in immunity.
Dölling (2022) Front Immunol. [43]	Multicenter Center (Germany)	Human autopsy samples	To investigate the neutrophil infiltration in cardiac tissue of patients with AMI	Nuclear HIF-1α is associated with prolonged neutrophil survival and enhanced oxidative stress in hypoxic areas of AMI.
Silvestre-Roig (2019) Nature [54]	Multicenter (Germany/Netherlands/ USA/France/Spain/Sweden)	Animal model Mouse models of atherosclerosis	To investigate chronic inflammation and its cellular and molecular mediators	Histone H4 binds to and lyses SMCs, leading to the destabilization of plaques. The neutralization of histone H4 prevents cell death of SMCs and stabilizes atherosclerotic lesions.
Talal et al. (2022) BMC Med [58]	Multicenter (Israel)	Human HD patients	To investigate mortality from bacterial infections in HD patients and NET role	Targeting NETosis in HD patients may reduce infections, minimize their severity, and decrease the mortality rate from infections in this patient population.

Abbreviations; AIPB, accelerated irrigation benefit program; ALI, Acute lung injury; AMI, acute myocardial infarction; BAP31, B-cell receptor associated protein 31; BM, bone marrow; CAD, coronary artery disease; CCL2, chemokine ligand 2; CCR2, C-C chemokine receptor type 2; CDK4, cyclin-dependent kinases 4; CHIP, Clonal hematopoiesis of indeterminate potential; Gαi2, g protein 2; GOF, gain-of-function; G-CSF, granulocyte colony-stimulating factor; HD, hemodialysis; HSPCs, hematopoietic stem and progenitor cells; JAK, Janus kinase; Ldlr−/−, low-density lipoprotein receptor knockout; MPNs, myeloproliferative neoplasms; NETome, neutrophil extracellular trap proteome; NLRP3, NOD-like receptor family, pyrin domain containing 3; PSGL-1, P-selectin glycoprotein ligand-1; RGS, Regulator of G protein signaling; SMC, smooth muscle cell; SREBP2, Sterol Regulatory Element-binding Protein-2; TET2, ten-eleven-translocation 2. * MISTRG acronym for the 7 modified genes in the genome of these mice: M-CSFh/h IL-3/GM-CSFh/h SIRPah/h TPOh/h RAG2−/− IL2Rg−/−.

**Table 2:** Characteristics of the included studies investigating the role of NET in arterial thrombosis.

First Author/Year Ref	Type of Study	Cohort	Aims	Finding
Fernandez et al. (2019) Nat Med [62]	Multicenter Center (USA/Sweden)	Human carotid artery plaques	To investigate the role of T cells and macrophages in carotid artery plaques of patients with recent stroke or transient ischemic attack compared to no recent stroke	In plaques from asymptomatic patients, T cells and macrophages were activated and displayed evidence of IL-1β signaling.
Zhao et al. (2022) J Hum Hypertens [68]	Multicenter Center (China/Sweden)	Human 4667 pts > 40 yrs	To study the relationship between NLR and the risk of CVD	When NLR was categorized into tertiles, participants in the top tertile had a significantly higher risk of CVDs (HR 1.61, 95% CI: 1.06, 2.44) and MI (HR 1.88, 95% CI: 1.09, 3.27) relative to those in the bottom tertile.
Locke et al. (2020) Sci Rep. [78]	Multicenter (UK)	Human blood donors	Whether formation of fibrinogen/fibrin-histone aggregates prevented cell death	Fibrinogen did not bind to or protect neutrophils stimulated with PMA. The role of fibrinogen in NETosis.
Noubouossie et al. (2017) Blood [89]	Multicenter (USA/Taiwan)	Human blood patients’ neutrophils	To investigate the mechanism leading to thrombosis promoted by intact NETs	Recombinant human histones H3 and H4 triggered TG in recalcified human plasma in a platelet-dependent manner. However, human intact NETs do not directly initiate or amplify coagulation in vitro.
Riegger et al. (2016) Eur Heart J [90]	Multicenter (Germany /USA/UK/ Netherlands/Belgium/ France/Spain/Polland)	Human 253 thrombus specimens	To evaluate thrombus specimens in patients with ST and presence of NET	Patients with ST revealed thrombus with leukocytes, particularly neutrophils in ST group. The presence of NETs supports their pathophysiological relevance.
Farkas et al. (2019) Thromb Res [91]	Multicenter (Hungary/Australia)	Human CAD 66 pts PAD 64 pts	To investigate the NET-related structural features of thrombi retrieved from different arterial localizations	NET content of thrombi was correlated with systemic inflammatory markers and with patients’ age. Evidence of NET-related variations in thrombus structure.
Laridan et al. (2017) Ann Neurol [95]	Multicenter (Belgium/Sweden)	Human 68 thrombus specimens	To investigate the presence of neutrophils and NETs in ischemic stroke thrombi	H3Ci higher in cardioembolic thrombi. Older thrombi contained significantly more neutrophils and H3Cit compared to fresh thrombi.
Shimony et al. (2010) Acute Card Care [110]	Single Center (Israel)	Human 16 randomly acute STEMI pts vs. 47 healthy individuals	To evaluate detection of CFD in patients with ST STEMI; to study correlation with established markers of necrosis and ventricular function	CFD levels were linked with the levels of established markers of myocardial necrosis but not with EF.
Langseth et al. (2020) PLoS One [111]	Multicenter (Norway)	Human 61 randomly STEMI pts	To investigate NETs’ associate to MF and IL 8 in STEMI patients with symptomatic acute HF	NETs’ components higher in acute heart failure and associated to myocardial function and interleukin 8 levels.
Langseth et al. (2020) Sci Rep [112]	Multicenter (Norway)	Human 956 cohort STEMI pts	To investigate association between circulating NETs-related components, clinical outcome, and hypercoagulability in STEMI	dsDNA levels were associated with increased all-cause mortality and with hypercoagulability in STEMI patients.
Helseth et al. (2016) Mediators Inflamm [113]	Single center (Norway)	Human 20 pts with STEMI vs. 10 pts with AP	To study infarct size and NETs’ markers in STEMI and AP	Higher levels of NETs in STEMI
Valles et al. (2017) Thromb Haemost [114]	Single center (Spain)	Human 43 pts with AIS	To evaluate NETs in the plasma of patients with acute ischemic stroke	At one-year follow-up, NETs were associated with severity and mortality. Relevant specific marker of NETs citH3 was higher and independently associated with AF and all-cause mortality. Significant role of NETs in the pathophysiology of stroke.
Hirose et al. (2014) PLoS One [115]	Multicenter (Japan)	Human 263 pts in ICU	To evaluate whether NETs and Cit-H3 were correlated with clinical and biological parameters	Crucial role of NETs in the biological defense against the dissemination of pathogens from the respiratory tract to the bloodstream in potentially infected patients.
Helseth (2019) Mediators Inflamm [116]	Multicenter Center (Germany)	Human 259 pts with STEMI	To explore circulating NET markers and associations to myocardial injury	dsDNA levels after STEMI were associated with myocardial infarct size, adverse left ventricular remodeling, and clinical outcome.
Langseth (2018) Eur J Prev Cardiol [117]	Multicenter (Norway)	Human 1001 pts with AP	To investigate the role of NETs’ markers, dsDNA, and myeloperoxidase-DNA in clinical outcome and hypercoagulability	Double-stranded DNA levels were significantly related to adverse clinical outcome after 2 years, but only weakly associated with hypercoagulability.
Martinod et al. (2016) Thromb Haemost [118]	Multicenter (USA)	Animal model WT vs. †NE (−/−) vs. SB1 vs. † NE (−/−) SB1 (−/−) mice.	Whether neutrophils from NE (−/−) mice have a defect in NETosis, similar to PAD4 (−/−)	Neutrophil elastase is not required for NET formation. NE (−/−) mice, which form pathological venous thrombi containing NETs, do not phenocopy PAD4 (−/−) mice in in vitro NETosis assays or experimental venous thrombosis.
Kim et al. (2017) J Immunol Res [119]	Multicenter (Korea)	60 pts in MHD	Whether NET formation was responsible for ESRD leading to higher incidence of CAD	Uremia-associated-increased NET generation may be a sign of increased burden of atherosclerosis.
Bang et al. (2019) Stroke [120]	Multicenter (Korea)	Human 138 randomly 38 pts cancer-related stroke 36 pts healthy-controls 27 pts cancer-controls (active cancer but no stroke) 40 pts stroke-controls (acute ischemic stroke but no cancer)	Whether NETs were increased in cancer-related stroke; whether higher NETs levels were associated with coagulopathy	Increased circulating DNA levels were associated with cancer-related stroke. NETosis was one of the molecular mechanisms of cancer-related stroke

Abbreviations; AF, atrial fibrillation; AIS, acute ischemic stroke; AP, stable angina pectoris; CAD, coronary artery disease; CFD, circulating cell free DNA; CVD, cardiovascular diseases; dsDNA, double-stranded DNA; end-stage renal disease (ESRD Il, interleukine; EF, ejection fraction; H, histone; H3Cit, citrullinated histone H3; HF, heart failure; MI, myocardial infarct; MF, myocardial function; MHD, maintenance hemodialysis; NE, neutrophil elastase, NET, neutrophil extracellular trap; NLR, neutrophils-to-lymphocyte ratio; PAD, peripheral artery disease; PAD4 (−/−), peptidylarginine deiminase 4; PMA, phorbol 12-myristate 13 acetate; ST, stent thrombosis; SB1, SerpinB1; STEMI, ST elevation myocardial infarction; TG, thrombin generation; WT, wilt type. † (−/−) Knock minu.

**Table 3:** Characteristics of the included studies investigating the role of NET in cardiovascular clinical biomarker applications.

First Author/Year Ref	Type of Study	Cohort	Aims	Finding
Mangold et al. (2015) Circ Res [93]	Single Center (Germany)	Human 111 pts with STEMI	To investigate relationships between CLS -NETs, bacterial components as triggers of NETosis, activity of endogenous deoxyribonuclease, ST-segment resolution, and infarct size	PMNs were highly activated in STEMI with NETosis at the CLS and coronary NET burden. Deoxyribonuclease activity was predictor of ST-segment resolution and myocardial infarct size.
Stakos et al. (2015) Eur Heart J [94]	Multicenter Center (Greece/Germany)	Human 18 pts with STEMI	To assess the in vivo importance of NETs during atherothrombosis	NETs by mean PMNs mediated thrombogenic signals during atherothrombosis.
Maugeri et al. (2020) Sci Rep [97]	Single center (IT)	Human	To assess the mechanism of platelets induced NETs	Activated platelets were related to HMGB1 in neutrophils. HMGB1 led to autophagy and NET generation.
Novotny et al. (2018) PLoS One [104]	Multicenter (Germany)	Human/animal model 81 human arterial thrombi retrieved mice with injury of carotid artery	To evaluate composition of arterial thrombi in mice compared to those of human patients with AMI	Inhibition of PAD was useful for the treatment of arterial thrombosis and to reduce NET generation.
Ducrox et al. (2018) Stroke [105]	Multicenter (France)	Human 108 pts with AIS	To determine the occurrence of NETs in thrombi retrieved during endovascular therapy. Impact on tPA-induced thrombolysis	The efficacy of a strategy involving an administration of DNAse 1 in addition to tPA was effective in the setting of AIS.
Cui et al. (2013) Cardiology [91]	Single center (China)	Human 137 pts with ACS 60 healthy individuals 13 pts with stable angina (SA)	To investigate cf-DNA concentrations in ACS and their relationship with clinical features	cf-DNA as a new valuable marker for diagnosing and predicting the severity of coronary artery lesions and risk stratification in ACS.

Abbreviations; ACS, acute coronary syndrome; AIS, acute ischemic stroke; AMI, acute myocardial infarction; cf-DNA, circulating cell free DNA; dsDNA, double-stranded DNA; HMGB1, high mobility group box 1 protein; NET, neutrophil extracellular trap; PMNs; polymorphonuclear neutrophils; SA, stable angina; STEMI, ST elevation myocardial infarction; tPA, tissue-type plasminogen activator.

## Data Availability

Not applicable.

## Supplementary Materials

The following supporting information can be downloaded at: https://www.mdpi.com/article/10.3390/biomedicines11010113/s1, Supplementary Table S1: Prisma checklist 2020. References [18,71,72,73,74,75] are cited in supplementary materials.
